# Supplementary material for: Effects of the acanthocephalan Polymorphus minutus and the microsporidian Dictyocoela duebenum on energy reserves and stress response of cadmium exposed Gammarus fossarum
Source: PeerJ. 2015 Oct 29;3:e1353. doi: 10.7717/peerj.1353 (PMC4631464; doi:10.7717/peerj.1353)

Supplemental figure S1. The infection percentage of microsporidians in *G. fossarum*. (A) males and (B) females. DICT: *Dictyocoela duebenum*, M505: *Microsporidium* sp. 505, MH: *Cystosporogenes* sp.

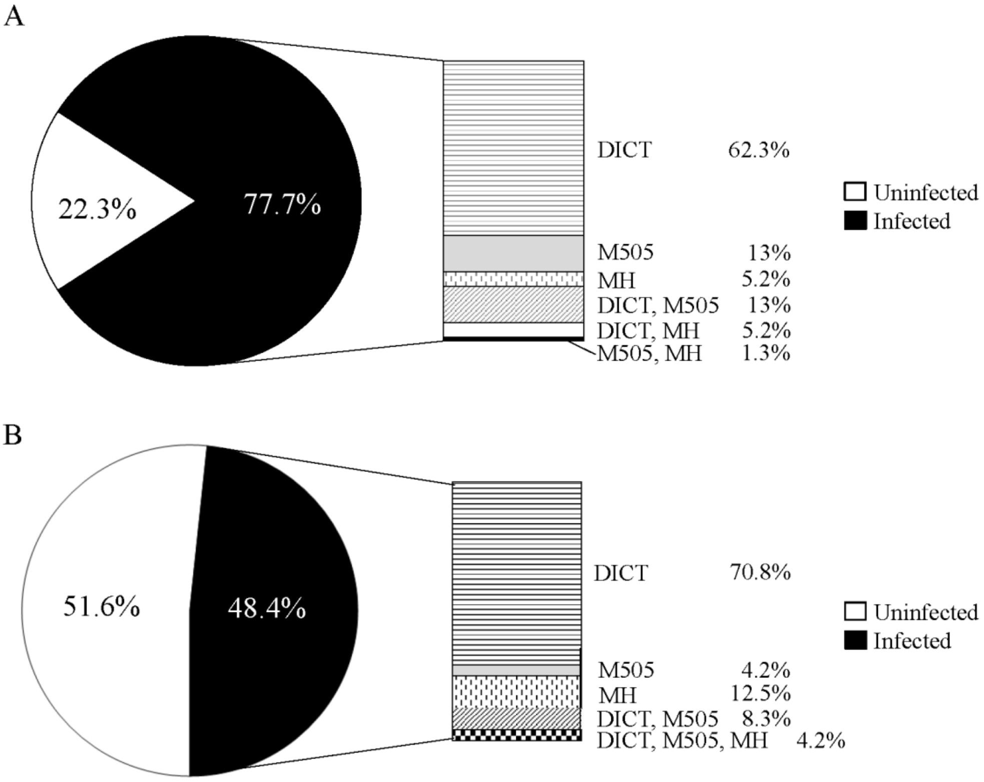

Supplement: Figure S1 [file peerj-03-1353-s001.pdf]
