# Supplementary material for: Effects of the acanthocephalan Polymorphus minutus and the microsporidian Dictyocoela duebenum on energy reserves and stress response of cadmium exposed Gammarus fossarum
Source: PeerJ. 2015 Oct 29;3:e1353. doi: 10.7717/peerj.1353 (PMC4631464; doi:10.7717/peerj.1353)
Supplement: Table S1 [file peerj-03-1353-s002.pdf]

Supplemental table S1. Prevalence of acanthocephalans and microsporidians in *G. fossarum* by sex.

Sex	Acanthocephala No. of examined *G. fossarum*	Acanthocephala No. infected (%)	Microspora No. of examined *G. fossarum*	Microspora No. infected (%)	Acanthocephala + Microspora No. infected (%)
Male	160	88 (55.0)	121	94 (77.7)	46 (38.0)
Female	139	55 (39.6)	91	44 (48.4)	10 (11.0)
Total	299[a]	143(47.8)	212[a]	138 (65.1)	56 (26.4)

[a] The difference between no. of individuals examined for microsporidians and acanthocepalans is due to the fact that the gammarids used for metal analysis could not be tested for microsporidians by PCR.
