# Supplementary material for: Effects of the acanthocephalan Polymorphus minutus and the microsporidian Dictyocoela duebenum on energy reserves and stress response of cadmium exposed Gammarus fossarum
Source: PeerJ. 2015 Oct 29;3:e1353. doi: 10.7717/peerj.1353 (PMC4631464; doi:10.7717/peerj.1353)
Supplement: Table S2 [file peerj-03-1353-s003.pdf]

Supplementary file 1: Output of GLS model analysis for the three biomarkers

Glycogen

```
Generalized least squares fit by REML
  Model: gylco ~ sex * expo * micro * acantho
  Data: dat
       AIC      BIC    logLik
  993.2456 1075.965 -464.6228

Coefficients:
                               Value Std.Error   t-value p-value
(Intercept)                103.36645   8.91557 11.593931  0.0000
sexM                       -18.84168  12.29646 -1.532285  0.1287
expoy                      -23.62171  10.78586 -2.190063  0.0309
microY                     -18.78795  10.90239 -1.723288  0.0880
acanthoY                   -14.67749  12.19726 -1.203342  0.2317
sexM:expoy                  51.97868  19.30738  2.692167  0.0083
sexM:microY                 35.74096  16.78268  2.129634  0.0357
expoy:microY                47.70546  17.46446  2.731574  0.0075
sexM:acanthoY               -0.18987  16.11392 -0.011783  0.9906
expoy:acanthoY              19.01391  14.82334  1.282701  0.2026
microY:acanthoY             23.16799  27.60219  0.839353  0.4033
sexM:expoy:microY          -82.02478  28.95110 -2.833218  0.0056
sexM:expoy:acanthoY        -35.47733  23.80081 -1.490594  0.1393
sexM:microY:acanthoY        -1.69905  33.75448 -0.050335  0.9600
expoy:microY:acanthoY      -26.13366  31.33908 -0.833900  0.4064
sexM:expoy:microY:acanthoY  34.42319  43.77138  0.786431  0.4335

Standardized residuals:
         Min           Q1          Med           Q3          Max
-1.908573640 -0.785069399 -0.006772025  0.657909605  2.405262368

Residual standard error: 23.95254
Degrees of freedom: 114 total; 98 residual
```

Lipids

```
> summary(lm.lip2)
Generalized least squares fit by REML
  Model: lipid ~ expo * micro * acantho * sex
  Data: dat
       AIC      BIC    logLik
  951.1571 1033.876 -443.5786

Coefficients:
                                Value Std.Error   t-value p-value
(Intercept)                  63.58453   6.01333 10.573927  0.0000
expoy                        -1.18338   7.51349 -0.157500  0.8752
microY                       43.78499  11.76413  3.721906  0.0003
acanthoY                    -16.30843   8.62651 -1.890501  0.0616
sexM                        -11.27344   9.75168 -1.156052  0.2505
expoy:microY                -52.36746  28.61114 -1.830317  0.0702
expoy:acanthoY               -0.92009  10.79655 -0.085221  0.9323
microY:acanthoY             -60.39784  25.45776 -2.372473  0.0196
expoy:sexM                  -13.59747  11.02799 -1.232995  0.2205
microY:sexM                 -16.43530  18.04145 -0.910975  0.3645
acanthoY:sexM                 2.93684  12.78824  0.229652  0.8188
expoy:microY:acanthoY        83.65573  36.74694  2.276536  0.0250
expoy:microY:sexM            57.33865  34.76548  1.649298  0.1023
expoy:acanthoY:sexM          19.85508  15.01409  1.322430  0.1891
microY:acanthoY:sexM         58.95679  30.26519  1.948007  0.0543
expoy:microY:acanthoY:sexM -100.82167  43.85624 -2.298913  0.0236

Standardized residuals:
       Min         Q1        Med         Q3        Max
-1.6080933 -0.6869587 -0.1507333  0.7022971  2.4488657
```

hsp70

```
Generalized least squares fit by REML
  Model: hsp ~ sex * expo * micro * acantho
  Data: dat
       AIC      BIC    logLik
  417.3562 498.3994 -176.6781

Coefficients:
                              Value Std.Error   t-value p-value
(Intercept)                3.236709  0.361425  8.955398  0.0000
sexM                       0.777097  0.798981  0.972610  0.3333
expoy                      0.482949  0.534040  0.904332  0.3682
microY                    -0.686953  0.521958 -1.316107  0.1914
acanthoY                   1.033026  0.573762  1.800441  0.0750
sexM:expoy                -0.157588  1.186726 -0.132792  0.8946
sexM:microY               -0.895088  0.982715 -0.910832  0.3647
expoy:microY               3.860698  1.507044  2.561768  0.0120
sexM:acanthoY             -2.058475  1.030372 -1.997797  0.0487
expoy:acanthoY            -1.213994  0.746596 -1.626039  0.1073
microY:acanthoY            0.935808  2.129076  0.439537  0.6613
sexM:expoy:microY         -0.571790  2.069833 -0.276249  0.7830
sexM:expoy:acanthoY        1.647666  1.501877  1.097072  0.2754
sexM:microY:acanthoY      -0.260797  2.362173 -0.110405  0.9123
expoy:microY:acanthoY     -3.831775  2.674886 -1.432500  0.1554
sexM:expoy:microY:acanthoY  2.262589  3.254903  0.695132  0.4887

Standardized residuals:
        Min          Q1         Med          Q3         Max
-1.79856503 -0.70711560 -0.03308062  0.79016412  1.81137055

Residual standard error: 2.015426
Degrees of freedom: 109 total; 93 residual
```
